# Nicotinamide Adenine Dinucleotide Does Not Improve Anesthetic Recovery in Rodents

**DOI:** 10.21203/rs.3.rs-4515123/v1

**Published:** 2024-06-20

**Authors:** Candida L Goodnough, July Montoya, Erica B Cartusciello, Erin L Floranda, Eric R Gross

**Affiliations:** Stanford University; Stanford University; Stanford University; Stanford University; Stanford University

## Abstract

Nicotinamide Adenine Dinucleotide (NAD^+^) is implicated in bioenergetics, DNA repair, and senescence. Depletion of NAD^+^ is associated with aging and neurodegenerative disease, prompting a growing interest in NAD^+^ supplementation. With rising over-the-counter use of NAD, understanding their impact on perioperative recovery becomes essential. This study investigates the effect of NADH, a common NAD^+^ precursor, on anesthesia in rodents. Baseline and post-anesthesia (1.5% isoflurane) open field and Y-maze activity were recorded in adult male and female C57/BL6 mice (n = 8–10/group). NADH (150 mg/kg, intraperitoneal) or vehicle (0.9% normal saline) were given at baseline or during anesthesia. The NADH-treated group exhibited a significant decrease in open-field activity relative to vehicle-treated. This diminished activity was reflected in reduced distance travelled and average velocity after emergence from anesthesia in the NADH-treated group. NADH treatment did not improve Y-maze performance after anesthesia as the number of visits to the novel arm was significantly decreased. This study demonstrates a potentially adverse impact of NADH on recovery from anesthesia. We revealed a depression in open-field activity and Y-maze performance with NADH supplementation, an indicator of cognitive recovery in rodents. The broad implications of NAD^+^ in aging are likely to shape supplementation trends, highlighting the importance of understanding the potential influence of administering NAD^+^ on anesthetic sensitivity and recovery.

## Introduction

Nicotinamide Adenine Dinucleotide (NAD^+^) is a critical coenzyme within all living cells regulating cellular bioenergetics, DNA repair, senescence, cell signalling, and mitochondrial homeostasis. Therefore, understanding the mechanisms underlying NAD^+^ homeostasis and how it relates to supporting brain health is important as NAD^+^ depletion is associated with normal and premature aging^1–3^. Interestingly, supplementation of NAD^+^ precursors reverses mitochondrial dysfunction and extends the life span for wild-type mice and mice with premature aging^4,5^.

As such, there is an increasing interest in using NAD^+^ supplements to reverse the hallmarks of brain aging^6^. As of April 2024, there were over 200 registered clinical trials utilizing NAD^+^ supplementation, largely related to preventing or treating aging-associated pathologies. There is now rising popularity of NAD^+^ and its precursors, including NADH, for over-the-counter use to treat dementia and for overall support of the aging brain. The global NAD market size is valued at just over USD 535 million in 2022 and will continue to grow over the next decade^7^. With increasing use of over-the-counter supplements to support brain health, the consequences of these supplements must be considered.

As the aging population expands, geriatric surgeries account for nearly half of all inpatient surgeries^8^. Of those geriatric surgeries, postoperative delirium affects 20–50% of patients after a major surgery^9,10^ and exerts substantial repercussions on a patient’s trajectory for recovery. Postoperative delirium is also linked to functional decline, prolonged hospitalization, institutionalization, and increased morbidity and mortality^10–19^. Thus, postoperative delirium underscores a significant clinical challenge. The treatment of post-operative delirium largely relies on risk-reductive strategies, including the avoidance of polypharmacy^20^, pre-operative pain control^21,22^, and avoiding prolonged fluid fasting^23^. However, few intraoperative interventions are effective against postoperative delirium^24^. Given the relative lack of interventions available to prevent delirium after surgery, an acute treatment given prior to emergence of anesthesia would be very appealing.

With the link of NAD supplementations improving brain aging, this poses a need to understand whether the acute administration of NAD^+^ precursors affect recovery from general anesthesia. The purpose of this study is to determine if NADH, a widely available and common additive to vitamins and supplements, impacts the recovery from general anesthesia in rodents.

## Results

A total of 52 rodents were used for the study. All rodents were included in the study and no rodents were excluded from data analysis. There were no rodent mortalities from this study. All data generated and analyzed during this study are included in this published article and are available from the corresponding author on reasonable request.

### NADH affects Baseline Open-field Activity

Without anesthesia, NADH slows open-field behavior in C57/BL6 mice (Fig. 2). NADH significantly decreased the active time in an open field for at least 30 minutes compared to vehicle (Fig. 2A, 28.4 ± 8%*** *vs* 79.7 ± 9%, respectively, *n* = 8 per group; ***p < 0.005 *vs* control). This was also reflected by reduced velocity travelled by the NADH treated group compared to vehicle (Fig. 2B, 0.34 ± 0.1cm/s**** vs 2.15 ± 0.3cm/s, respectively, *n* = 8 per group; ****p < 0.001 vs control) and total track length (Fig. 2C, 636.5 ± 237cm** vs 3591 ± 676cm, respectively, *n* = 8 per group; **p < 0.01 *vs* vehicle).

### NADH Slows Open-field Behavior after Emergence from General Anesthesia

Open field behavior after emergence from general anesthesia was quantified. In addition, representative open-field tracking images in the vehicle and NADH treated mice after the emergence of anesthesia were obtained (Fig. 3A and 3B). After emergence from anesthesia, the NADH treated group was less active compared to vehicle (Fig. 3C). When normalized to baseline open-field activity, the NADH treated group was significantly less active compared to vehicle in the 30 minutes following emergence from general anesthesia (Fig. 3D, 24.4 ± 7cm/s** vs 61.4 ± 6cm/s, respectively, *n* = 8 per group; **p < 0.01). The distance travelled and the average velocity were significantly decreased in the NADH treated group after emergence (Fig. 3E and 3F, distance travelled: 946 ± 448cm* *vs* 2501 ± 325cm, and average velocity: 0.52 ± 0.25cm/s* *vs* 1.39 ± 0.18cm/s, *n* = 8 per group, *p < 0.05). NADH treatment did not affect emergence, as the time to regain the righting reflex was no different between the NADH treated or vehicle untreated groups (Fig. 3G, 4.9 ± min *vs* 4.0 ± min, respectively, *n* = 8 per group).

### NADH does not improve Y-maze performance after anesthesia

The number of visits to the novel arm was significantly decreased in the NADH treated group after emergence from anesthesia when compared to number of visits prior to anesthetic administration (Fig. 4A, NADH group: 11 ± 3*** vs 34 ± 5, respectively, n = 10 per group, ***p < 0.005). In contrast, there was no effect of anesthesia on the novel arm visits in the vehicle-treated group when comparing number of visits after emergence with respect to number of visits prior to anesthetic administration (Fig. 4A, vehicle group: 25 ± 3 vs 24 ± 3, respectively, n = 10 per group). There was no significance difference between novel arm visits at baseline between vehicle and NADH treated groups. The number of visits to the alternate arm were also significantly decreased after anesthesia in the NADH treated group as compared to prior to anesthesia, but there was no difference in alternate arm visits between the NADH and vehicle treated groups after anesthesia (Fig. 4B, NADH group: 20 ± 3 *vs* 6 ± 2***, respectively, vehicle group: 10 ± 2 *vs* 14 ± 2, n = 10 per group, ***p < 0.005). Although there were fewer novel arm visits in the NADH treated group, there was no significant difference in the novel arm visit duration between NADH and vehicle before or after the anesthetic (Fig. 4C, NADH group: 248 ± 33sec *vs* 255 ± 69sec, vehicle group: 278 ± 16sec *vs* 268 ± 30sec, n = 10 per group). Similar to the open-field activity, the NADH treated mice had a slower average velocity participating in the Y-maze as compared to vehicle treated mice after anesthesia (Fig. 4D, 2.6 ± 0.5*cm/s *vs* 3.9 ± 0.3cm/s, respectively, n = 10 per group, *p < 0.05).

## Discussion

Aside from using NAD + in the prevention and treatment of Alzheimers Disease and other forms of dementia^6^, there is a newfound interest in using NAD + to prevent post-operative delirium. However, we find that NADH, a common over-the-counter supplement in adults, decreases the activity of rodents alone and following the emergence from general anesthesia. This was reflected by a decrease in open-field velocity, track length, and percent active time in mice that received NADH. In addition, NADH decreased the number of novel arm visits in a post-anesthesia Y-maze trial compared to baseline, indicating a deficit in short-term spatial memory performance. To our knowledge, this is the first study to demonstrate that acute NADH administration slows activity and special memory performance in rodents after isoflurane-induced general anesthesia.

NAD^+^ depletion is a hallmark feature of normal aging and in neurodegenerative diseases, including Parkinson’s and Alzheimer’s diseases^2,25,26^. Therefore, the use of NAD^+^ and its precursors, including NADH, have garnered significant attention for its potential to minimize the impact of brain aging. Zhu et al. showed that there is an age-dependent decrease in NAD + in the brains of healthy adults^3^, which was in agreement with the observed age-dependent reduction of NAD + in the hippocampus of aged mice^27^. The age-related reduction in NAD + may be related to increased consumption by Poly (ADP-ribose) polymerase (PARPs) and CD38, which is in parallel with mitochondrial dysfunction in a sirtuin-dependent manner^2,28^. Importantly, NAD^+^ augmentation increases resistance to oxidative stress, increases neurogenesis, and improves neuronal plasticity and cognitive function in aged rodent models^4,29^. The broad implications for NAD^+^ and its role in healthy and pathologic aging have a major impact on NAD^+^ supplementation in the adult population. It is important to recognize the potential impact NAD^+^ has on recovery after anesthesia given its close link to neuronal plasticity and bioenergetics.

There are very few intraoperative treatments that prevent post-operative delirium. A recent meta-analysis, including 4 randomized control trials and 2 observational studies, demonstrated a 45% reduction in delirium risk with perioperative melatonin^30^. Another meta-analysis suggested a trend towards reduced postoperative delirium in cardiac surgery patients receiving intraoperative dexamethasone (8mg or 1mg/kg), however the safety of such high doses and its effect on non-cardiac surgeries remains unclear^31^. This study showed that the open-field and Y-maze activity, indicators of cognitive recovery in rodents, were less in rodents receiving acute NADH administration relative to vehicle. NADH, a common NAD + precursor, may not improve post-anesthetic cognitive recovery despite its promising use to prevent and treat dementia.

Our results need to be considered within the context of potential limitations. NAD^+^ was acutely administered just prior to emergence and may not necessarily be representative of how chronic NAD supplementation may impact recovery from general anesthesia. This may explain why our study differs from the improvement in cognitive performance seen by others with chronic NAD administration in rodents^29^. Regardless, acute administration of NADH altered rodent behavior which underscores that there are differences in the effect of acute and chronic administration of NAD. Moreover, this leads to question whether patients taking NAD who will have planned surgery with anesthesia may benefit from discontinuing NAD supplements just prior to surgery. Further, although the mechanism through which NADH slows open-field activity is not known, this interesting observation that NADH decreases activity and spatial recognition after anesthesia warrants further investigation.

This study explores the effects of NADH, a common over-the-counter NAD + precursor, on cognitive and locomotor recovery after anesthesia in rodents. Despite the interest in NAD + for its potential benefits in brain aging, our findings indicate that acute NADH administration decreases rodent activity and impairs short-term spatial memory performance following general anesthesia. The mechanisms underlying these effects should be evaluated in future research, as well as the potential benefits or risks of discontinuing NAD supplements in the perioperative period.

## Methods

Procedures and protocols were approved by the Animal Care and Use Committee at Stanford University under AAPLAC #31510 (Stanford, CA, USA) and all methods were performed in accordance with their guidelines and regulations. In addition, the study was conducted in accordance with ARRIVE guidelines.

Twelve- to sixteen-week old male and female C57/BL6J mice (Jackson Labs, Sacramento, CA) were used. Based on an a priori power analysis (a = 0.05 and 80% power), we determined that n = 8 mice were needed for the open-field behavior studies, and n = 10 were needed for Y-maze behavioral studies.

The drugs used in the study included NADH (150 mg/kg^32^, intraperitoneal, Thermo Fisher Scientific, Catalog No. AAJ6163803, Waltham, MA) and vehicle (0.9% normal saline). To determine how NADH impacted recovery from general anesthesia, a sub-set of rodents received isoflurane (1.5%, inhalational, VetOne, Boise, Idaho) prior to NADH or vehicle administration.

**Figure 1:**
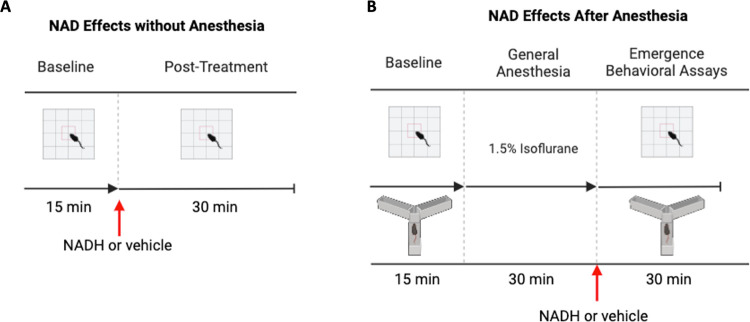
Experimental protocols. (A) After collection of baseline open-field data, NADH (150mg/kg, intraperitoneal) or 0.9% normal saline vehicle (0.1 mL, intraperitoneal) were given. Mice were returned for open-field observation for 30 minutes after treatment. (B) To determine the impact of NAD supplementation on general anesthesia, baseline open-field and Y-maze activity for 15 minutes was recorded. Mice were then anesthetized for 30 minutes with 1.5% isoflurane. Just prior to emergence, mice were treated with NADH (150mg/kg, intraperitoneal) or 0.9% normal saline vehicle (0.1 mL, intraperitoneal). After the return of the righting reflex, open-field and Y-maze were separately recorded for 30 minutes to assess anesthetic emergence activity.

**Figure 2:**
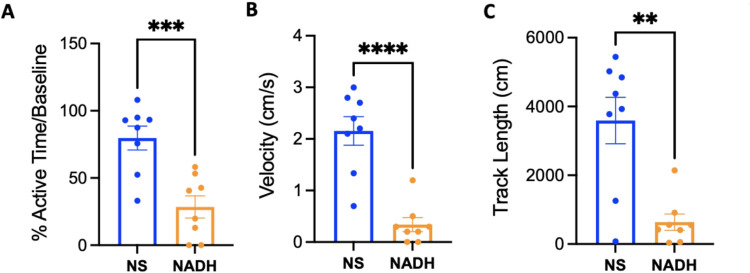
Impact of NADH on rodent activity without anesthesia. (A) The percent time active for vehicle or NADH treated groups normalized to the pre-treatment baseline activity. (B) The average velocity (cm/s) of vehicle or NADH treated activity in an open-field averaged over 30 minutes. (C) The total track length (cm) traveled in the open-field for 30 minutes after treatment with vehicle or NADH. NADH; nicotinamide adenine dinucleotide hydrogen, IP; intraperitoneal. n=8/group. **p<0.01, ***p<0.005, ****p<0.001, student’s t-test.

**Figure 3:**
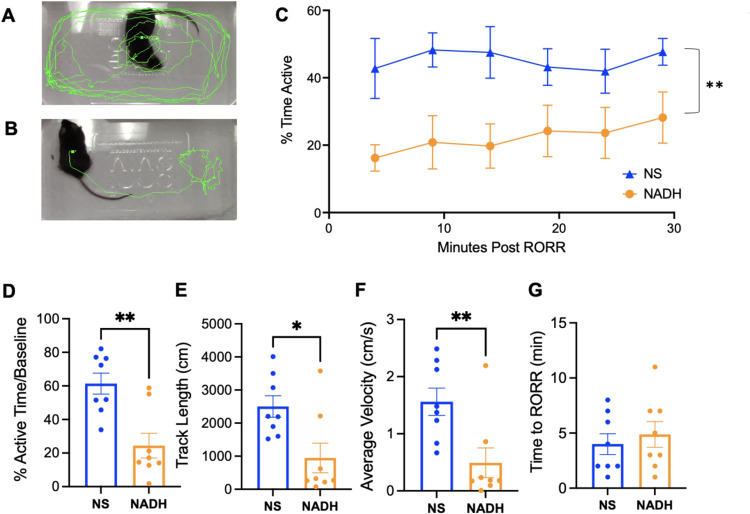
Impact of NADH on post-anesthetic rodent activity (A and B) Representative open-field tracking images for the first two minutes after emergence from anesthesia in (A) vehicle and (B) NADH-treated mice. (C) Time course representing the percent time that the vehicle treated or NADH treated mice were active in an open-field for 30 minutes after the emergence of anesthesia. (D) The percent active time normalized to the pre-anesthetic baseline activity in vehicle or NADH treated groups averaged over the first 15 minutes. (E) The total track length (cm) traveled in the open-field for 30 minutes after treatment with vehicle or NADH. (F) The average velocity (cm/s) of vehicle or NADH treated activity in an open-field averaged over 30 minutes. (G) The time to regain the righting reflex after discontinuation of isoflurane. NADH; nicotinamide adenine dinucleotide hydrogen, IP; intraperitoneal, RORR; return of righting reflex. n=8/group. *p<0.05, **p<0.01, one-way ANOVA (C) or student’s t-test (D-F).

**Figure 4:**
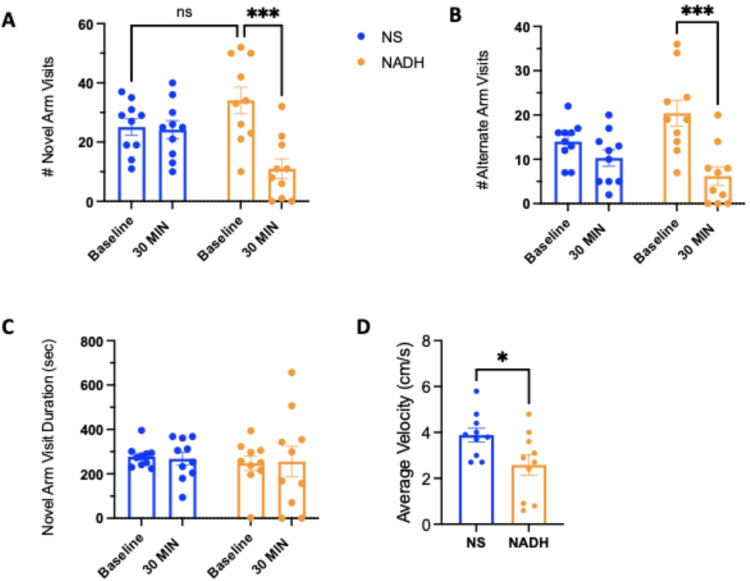
Impact of NADH on post-anesthetic short-term spatial memory in rodents. (A) The number of visits to the novel arm of the Y-maze at baseline and 30 minutes after emergence from general anesthesia in vehicle and NADH treated groups. (B) The number of visits to the alternate arm at baseline and 30 minutes after emergence from general anesthesia in vehicle and NADH treated groups. (C) The total duration spent in the novel arm at baseline and 30 minutes after emergence from general anesthesia in vehicle and NADH treated groups. (D) The average velocity in all arms of the Y-maze for the duration of the experiment 30 minutes after emergence from general anesthesia in vehicle and NADH treated groups. NADH; nicotinamide adenine dinucleotide hydrogen. n=10/group. *p<0.05, ***p<0.005, one-way ANOVA (A-C) or student’s t-test (D).

The first sub-set of mice received NAD without general anesthesia while a different sub-set of mice received NAD with general anesthesia (Fig. 1). For mice receiving NAD without general anesthesia, baseline and post-treatment open field activity was recorded (Fig. 1A). After 15 minutes acclimation, the baseline open field activity was recorded for 15 minutes in all mice. Mice were then given NADH or vehicle and returned to the open field to continue recording for 30 minutes post-treatment.

For mice receiving NADH with anesthesia, the baseline activity was recorded prior to anesthesia and the treatment was given prior to emergence from anesthesia (Fig. 1B). After 15 minutes acclimation, the baseline open field activity was recorded in all mice. Mice were then anesthetized in an induction chamber on a heating pad with 1.5% isoflurane for 30 minutes. The mice then received NADH or vehicle just prior to emergence and returned to the open field to continue recording for 30 minutes post treatment. In a separate cohort of mice, the Y-maze performance was assessed at baseline and post treatment.

The behavioral assays used to assess general locomotor activity and short-term memory included open field activity and Y-maze^33^. For open field activity, activity was recorded after the return of the righting reflex for a period of 30 minutes to assess recovery after anesthesia. Open-field activity was analyzed by a blinded observer using Biobserve (Bonn, Germany) tracking software. The percent time a mouse was active in an open-field (defined as movement with a minimum velocity of 0.5 cm/s) was recorded and normalized to the pre-treatment baseline activity. Y-maze activity was recorded 30 minutes after the emergence from anesthesia with NADH or vehicle. After recovery for 30 minutes after anesthesia, mice were placed in the Y-maze with 2 arms open (start and alternate arms) for a period of 10 minutes and returned to their cage for 30 minutes. Mice were then returned to the Y-maze with all 3 arms open, and activity was recorded for 10 minutes. The Y-maze activity was analyzed by a blinded observer using Biobserve (Bonn, Germany) tracking software.

GraphPad Prism (Boston, MA) was used for statistical analysis. Data are expressed as mean ± SEM. An unpaired student’s t test was used to compare two groups while for multiple time points ANOVA with Bonferroni correction was used. Statistical significance was defined as p < 0.05.

## Data Availability

All data generated and analyzed during this study are included in this published article and are available from the corresponding author on reasonable request.
